# Encapsulation of Low-Molecular-Weight Drugs into Polymer Multilayer Capsules Templated on Vaterite CaCO_3_ Crystals

**DOI:** 10.3390/mi11080717

**Published:** 2020-07-24

**Authors:** Jack Campbell, Georgia Kastania, Dmitry Volodkin

**Affiliations:** School of Science and Technology, Nottingham Trent University, Clifton Lane, Nottingham NG11 8NS, UK; jack.campbell@ntu.ac.uk (J.C.); georgia.kastania2016@my.ntu.ac.uk (G.K.)

**Keywords:** polyelectrolyte multilayer capsules (PEMCs), calcium carbonate, release kinetics, ibuprofen, doxorubicin

## Abstract

Polyelectrolyte multilayer capsules (PEMCs) templated onto biocompatible and easily degradable vaterite CaCO_3_ crystals via the layer-by-layer (LbL) polymer deposition process have served as multifunctional and tailor-made vehicles for advanced drug delivery. Since the last two decades, the PEMCs were utilized for effective encapsulation and controlled release of bioactive macromolecules (proteins, nucleic acids, etc.). However, their capacity to host low-molecular-weight (LMW) drugs (<1–2 kDa) has been demonstrated rather recently due to a limited retention ability of multilayers to small molecules. The safe and controlled delivery of LMW drugs plays a vital role for the treatment of cancers and other diseases, and, due to their tunable and inherent properties, PEMCs have shown to be good candidates for smart drug delivery. Herein, we summarize recent progress on the encapsulation of LMW drugs into PEMCs templated onto vaterite CaCO_3_ crystals. The drug loading and release mechanisms, advantages and limitations of the PEMCs as LMW drug carriers, as well as bio-applications of drug-laden capsules are discussed based upon the recent literature findings.

## 1. The Development of Polyelectrolyte Multilayer Capsules (PEMCs) as Drug Delivery Vehicles

The recent development of drug delivery systems such as liposomes, micelles and polymeric micro-/nanoparticles and containers plays a vital role in the confrontation of several diseases (such as cancers) and for use in vaccinations. Such systems have the ability to protect, safely deliver and release drugs (i.e., antibiotics, chemotherapeutic drugs, etc.), proteins, RNA stimuli, and others at a targeted biological site in a controlled manner [1,2,3].

Some porous inorganic materials that are biologically stable and exhibit a controlled release property, namely host/guest drug delivery systems, are attractive as alternative delivery vehicles. Examples of these systems are porous silica nanoparticles [4], silica-calcium phosphate composites [5], porous hydroxyapatite [6], and vaterite calcium carbonate crystals [7,8]. Despite the fact that these inorganic materials can encapsulate such a cargo, an initial burst release mode (where the vehicle initially releases the majority of its cargo rapidly) has been reported in many cases [4,9].

The layer-by-layer (LbL) assembly process is a powerful tool for developing two-dimensional polymeric films [10] and three-dimensional particles [11]. Such systems can effectively host proteins and other (bio)molecules of different nature, serving as high-level mimics of the extracellular matrix in 2D and 3D [12,13,14,15,16]. The utilization of this approach has assisted in the recent developments within the encapsulation of drug-loaded microparticles and in the reduction of release rate and the suppression of the initial burst release of these systems [17]. Popular drug delivery carriers developed as of recent are thin-walled PEMCs with a pre-designed geometry and size. PEMCs are fabricated by the alternate deposition of oppositely charged polyelectrolytes onto decomposable core templates—resulting in a core-shell complex formation. This is followed by the elimination of the core template, leaving a free-standing polymeric shell or particle i.e., a PEMC [17,18,19]. Not only solid decomposable cores have been used for formation of advanced multilayer structures, but also soft particles such as protein aggregates [20,21] and biological cells [22].

Polymeric capsules have shown to be prime candidates for the delivery of biological cargo. This is due to their finely tunable properties (e.g., shell thickness and permeability [23], as well as capsule shrinkage and swelling [24,25]) which can be easily altered or induced via changes in multilayer build-up conditions and by the introduction of external stimuli after multilayer build-up, such as changes in pH [26,27], ionic strength [28], and temperature [29,30,31]. The fabricated PEMCs can hold diameters ranging from the nano- to the micro-meter scale. This is dependent upon the size of the initial template utilized, as well as whether the formed capsule has been exposed to external stimuli. Such reduction in PEMC size has been demonstrated by Köhler et al. via subjecting pre-formed synthetic-(poly(styrene)sulfonate (PSS)/poly(diallyldimethylammonium chloride) (PDADMAC)) [32] and bio-capsules (dextran sulfate (DS)/poly-l-arginine (PARG)) [33] to heat treatment in order to control capsule permeability and size of carriers [34]. Moreover, the nature and blend of the polymers constituting the multilayers must be considered when taking into account the final application of the PEMCs. For instance, synthetic polymers offer high control over chemical quality (i.e., purity) and abundance, hold higher flexibility than natural counterparts, and offer chemical inertness in multiple environments, however, they are often non-biodegradable and toxic. Natural polymers on the other hand are highly biocompatible, biodegradable, and offer bioactivity (e.g., cell recognition), but are intrinsically polydisperse and typically hold only small pH and ionic strength working windows (reviews [35,36] and references therein). These easily tailored properties have many implications for PEMCs in the design of drug delivery vehicles.

The aforementioned vaterite CaCO_3_ crystals are widely used as sacrificial cores for the fabrication of PEMCs [37,38,39] as well as larger tailor-made polymer based structures like porous polymer scaffolds [40,41,42,43]. The vaterite polymorph belongs to a family of naturally derived minerals [44], but can be readily produced from low-cost precursor salts (e.g., sodium carbonate and calcium chloride) at either the micro- or nanoscale [45,46]. Moreover, they are eco-friendly and highly biocompatible, and may be readily decomposed under mild conditions (e.g., at slightly acidic pH below 7 or in the presence of chelating agents such as ethylenediaminetetraacetic acid (EDTA) or citric acid). The crystals are mesoporous materials and therefore exhibit high loading capacities; allowing them to effectively host and protect both small compounds like drugs [47], nanoparticles [48,49], and larger biomacromolecules such as proteins [50,51,52], hormones [53], and enzymes [54,55]. Interestingly, the loading of large charged macromolecules such as proteins is governed by both the protein affinity to the CaCO_3_ surface and inter-protein interactions in the presence of CaCO_3_ [56,57]. The loading procedure can be done at fully biocompatible conditions at pHs close to the physiological one that preserves protein bioactivity [58]. The PEMCs formed onto the vaterite crystals may be of either hollow or matrix type depending upon diffusion of polymers during the LbL deposition that is governed by polymer interactions and properties [17,43].

Despite a significant progress and obvious advantages in the utilization of PEMCs templated onto the vaterite crystals for loading and controlled release of large bioactive molecules, low-molecular-weight (LMW) drugs have also been recently employed for encapsulation aiming at advanced drug delivery. However, this requires special approaches due to high permeability of polymer multilayers for small compounds, limiting retention and controlled/sustained release of LMW drugs. This topic will be addressed in the following chapters.

## 2. The Loading of LMW Drugs into PEMCs

### 2.1. LMW Drugs

**Figure 1:**
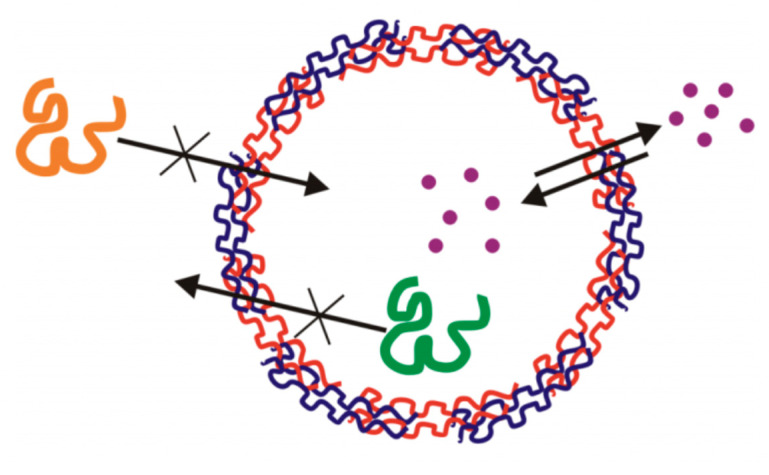
Schematic representation of the permeability of the capsule shell to small, low molecular weight drugs and water molecules (purple), as well as larger, impermeable molecules (orange/green). This schematic is taken with permission from Reference [59], copyright© 2011, Elsevier.

Drugs that are used for the treatment of several diseases are typically small, and hence possess a low molecular weight; approximately smaller than 1–2 kDa. Such small drugs can readily penetrate both the membrane of diseased cells and the multilayer capsules; where larger macromolecules may be impermeable to capsule shell, as illustrated in Figure 1 [59]. The encapsulation of the drugs can be achieved either via physisorption [50,60] or co-synthesis [61,62,63,64,65] (i.e., post- or pre-loading, respectively).

**Figure 2:**
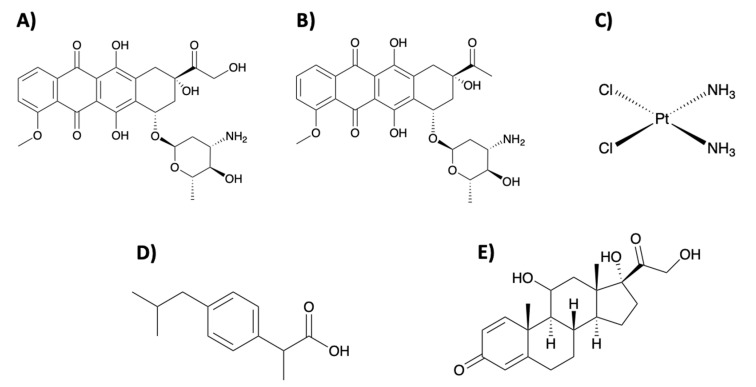
Molecular structures of (**A**) doxorubicin, (**B**) daunorubicin, (**C**) cisplatin, (**D**) ibuprofen, and (**E**) prednisolone.

Some examples of such drugs include the extensively studied doxorubicin (DOX) and *cisplatin* anticancer drugs, which are effective for several types of cancers, as well as daunorubicin (DNR), which is used as treatment of acute myeloid leukemias. Ibuprofen (IBU), which is used as an anti-inflammatory drug, has seen much recent work, as well as prednisolone, a corticosteroid which is used for the treatment of many conditions including rheumatoid arthritis. The structures of such drugs are seen in Figure 2.

### 2.2. Mechanism of LMW Drug Loading

For the loading of small positively charged and water-soluble drugs, two strategies have recently attracted awareness; these are the “spontaneous” and the “charge-controlled attraction and repulsion” processes [66,67,68,69,70,71,72]. In the case of the spontaneous deposition strategy, the pre-loading of a negatively charged matrix is crucial for the permission of a positively charged drug to be incorporated within the final fabricated capsules. The pre-loading step includes pre-encapsulation of the matrix material (typically a polyelectrolyte); in which case, this material must have a high affinity to the core (i.e., lentinan [73] or heparin [74]), as well hold some affinity to the drug of interest. This is then followed by the elimination of the core, where the pre-loaded polyelectrolyte remains inside the formed capsule (this step is crucial for both methods). However, in some cases during this matrix-loading, after the pre-loading of a negatively charged polyelectrolyte and the removal of the core, a part of the negatively charged polyelectrolyte is entangled within the multilayers; some of which is paired up with the excess adsorbed polycation. This is the ‘charge-controlled attraction repulsion’ effect. This, in effect, allows permeation of positively charged drugs through the capsule shell, whilst simultaneously repelling negatively charged drugs [72]. In addition, studies have shown that positively charged drugs can be automatically loaded effectively, and accumulation of the molecule of interest into the prefabricated capsules is ten to hundreds of times higher than the feeding drug concentration [66,67,68,69,70]. A similar method has been used to selectively encapsulate both positively and negatively charged drugs into PEMCs within polypeptide films, depending upon the charge of the pre-loaded matrix [75].

**Figure 3:**
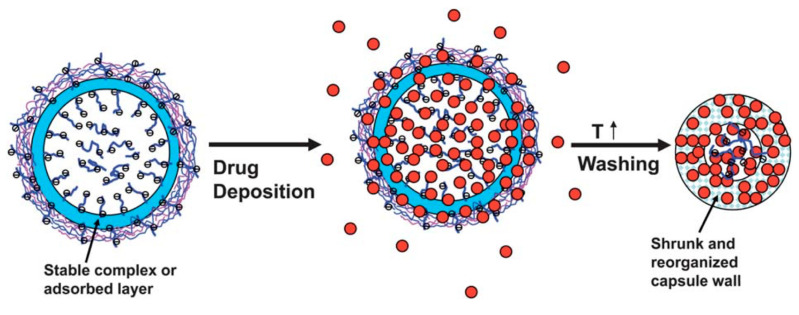
Schematic illustration of the loading of low-molecular-weight (LMW) drugs into PSS-loaded (PDADMAC)/PSS)_5_-PSS multilayer capsules through a combination of the spontaneous deposition process and heat-driven shrinkage of capsules. The blue layer refers to the adsorbed layer, which is formed from the first PDADMAC layer and free PSS polyelectrolyte during the crystal dissolution process. The pale blue rhombus refers to the shrunk and re-organized capsule shell. This schematic is taken with permission from Reference [76], copyright© 2011, Royal Society of Chemistry.

Many studies have been performed in order to understand the loading mechanism of drugs into such capsules. A popular matrix material that has seen much use is poly(styrene)sulfonate (PSS), a negatively charged polyelectrolyte, which therefore has high affinity to positively charged drugs. Tong et al. (2011) [76] developed hollow capsules consisting of five bilayers of PDADMAC and PSS polyelectrolytes, templated onto PSS-doped CaCO_3_ crystals in order to examine the loading mechanism of the positively charged DOX. The DOX was accumulated into the capsules’ interior, and this is due to the favored interaction between the negatively charged PSS and positively charged DOX molecules. The loading of DOX is illustrated in Figure 3. In order to ensure the complete entrapment within the capsule lumen, the capsules were shrunk via heat-treatment. Heating multilayer systems typically results in the annealing of the polymer multilayers [77,78] and the closure of pores and defects present in the capsule wall due to compaction [79], resulting in an impermeable wall for DOX. This method has shown to dramatically reduce capsule permeability and enhance retention after shrinkage.

Furthermore, Balabushevich et al. (2019) [80] demonstrated the uptake of DOX within mucin-doped CaCO_3_ vaterite crystals, reporting a significant increase in the efficiency of loading DOX compared to that of bare CaCO_3_, due to DOX-mucin electrostatic interactions. Similarly, polyanionic carboxymethyl cellulose (CMC) has also seen use as the matrix material pre-loaded within the CaCO_3_ cores. CMC holds carboxylic groups, which under certain conditions will ionize and become negatively charged. Hence, with the same mechanism as PSS-doped cores, the positively charged anticancer drugs can bind to the negative charges of the deprotonated carboxylic groups through electrostatic interactions, and eventually be accumulate inside the CMC-doped multilayer capsules, as demonstrated in chitosan (CS)/alginate (ALG) multilayer systems [81,82].

The encapsulation of hydrophobic drugs within PEMC and CaCO_3_ interiors has also been demonstrated via alternate means. Cyclodextrins (CDs) are ideal candidates for this and have been previously utilized; containing a hydrophobic interior and hydrophilic exterior, they are ideal hosts for hydrophobic entities. CDs have seen use via modifying multilayers with CD entities [83]; for instance, hyaluronic acid (HA) modified with CDs has been paired with poly-l-lysine (PLL) to form PEMCs, using CaCO_3_ vaterite as a sacrificial core. The hydrophobic anticancer drug, paclitaxel, was pre-complexed with the CD-modified HA prior to the LbL coating and CaCO_3_ dissolution. Hence, after PEMC formation, the paclitaxel remained within the capsule shell [84]. Polymeric CDs may also be pre-loaded into the CaCO_3_ crystal matrix via co-synthesis [85,86,87] with pre-complexed drug or as empty CDs for drug post-loading. Moreover, polymeric CDs have also been used as constituents of multilayers forming PEMCs [88].

### 2.3. Factors Influencing Drug Loading

Multiple factors can have profound effects upon the efficiency and extent of drug-loading. Factors such as the matrix content within the crystals, the drug incubation temperature, as well as ionic strength, pH, drug feeding concentration, and the number of layers constituting the multilayer shell are observed to have a dramatic impact upon the drug accumulation performance of the capsules. One can expect the extent of the pre-loaded matrix mass content of the CaCO_3_ crystals to influence the adsorption of the drug of interest. For instance, Liang et al. (2013) [65] demonstrated the loading of heparin into CaCO_3_ crystals via co-synthesis, followed by the uptake of DOX via physisorption. The larger the mass content of heparin within the crystals, the greater the DOX uptake, likely due to the electrostatic interaction between the two molecules. This is similar to the work of Shi et al. (2019) [51], who reported the increased uptake of lysozyme with increased heparin content. Potentially a number of biologically relevant small molecules (e.g., lipids forming lipid-protein complexes [89,90,91]) can be employed to be loaded into the vaterite crystals that serve as a universal carrier due to its mesoporous structure.

**Figure 4:**
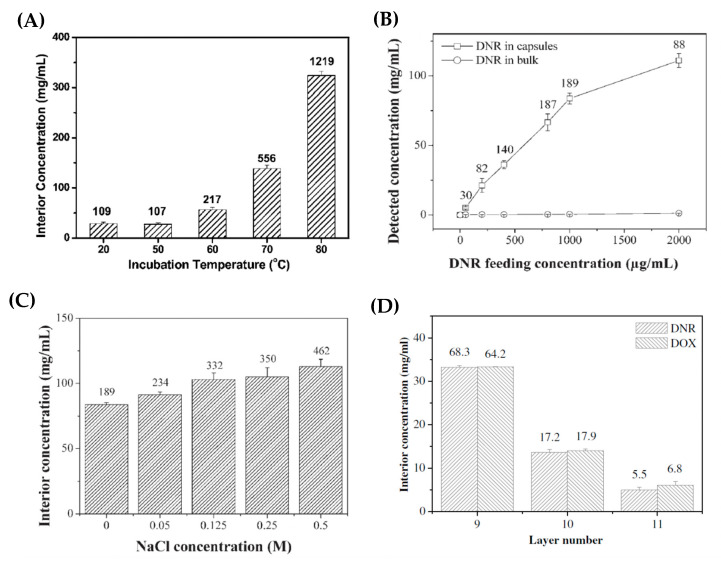
(**A**) The effect of incubation temperature upon DOX uptake into (PSS/PDADMAC)_5_ capsules; (**B**) the effect of the DNR feeding concentration upon the uptake into ALG)/CS)-based capsules; (**C**) the effect of ionic strength (NaCl concentration) upon the loading of DNR into ALG/CS-based capsules; and (**D**) the effect of the number of layers upon the uptake of both DOX and DNR within poly(allylamine hydrochloride) (PAH)/PSS-based capsules. The values embedded in the figures represent the concentration ratios between the capsule interiors and the bulk. Figure A taken with permission from Reference [76], copyright© 2011, Royal Society of Chemistry. Figures B and C taken with permission from Reference [92], copyright© 2007, John Wiley and Sons. Figure D taken with permission from Reference [93], copyright© 2006, Elsevier.

By varying the particle-drug incubation temperature, it was revealed that, as the incubation temperature increased, the deposition concentration of DOX inside the interior of PSS-doped capsules was increased dramatically—as illustrated in Figure 4A. The ratio of capsule interior DOX concentration to the bulk DOX concentration increases from ~100 (for pre-heat treated capsules) to >1000 (for heat treated capsules at 80 °C); the authors attribute this ratio increase to the PEMC shrinkage phenomena (Figure 3) [76], during which the capsule wall becomes more compact and, perhaps, the DOX is better retained after washing stages compared to original capsules. In addition, Han et al. (2008) [92] observed a similar response of CMC-doped CS/ALG-based capsules to an increase in temperature upon the loading of DNR; with an increase in local DNR concentration within the capsules as the temperature is increased. However, in this case, it was assumed that the CMC swelled about heating, allowing for further DNR accumulation into the capsules.

The drug loading performance of the capsules can also be greatly affected by the feeding concentration of the drug [76,81,82,93]. Multiple studies have revealed that, as the feeding concentration is increased, both non-linear and linear relationships are observed for the increase of drug concentrations in the interior of capsules and in the bulk, respectively. For instance, the absolute amount of loaded DOX increases with increasing DOX feeding concentration, whilst encapsulation efficiencies decreased gradually. At lower feeding concentrations, the loading may not be thermodynamically saturated and equilibrated, hence, this demonstrates how effective the spontaneous deposition process can be when the matrix material holds high affinity to the drug of interest, as large amounts of the drug may still be loaded even at low feeding concentrations. Furthermore, it is reported that an increased amount of drug may be loaded into the interior of the capsules observed at higher ionic strengths (Figure 4C) [92,93]. This is likely due to the increased multilayer porosity and fluidity at higher ionic strengths, allowing for less hindered drug diffusion.

**Figure 5:**
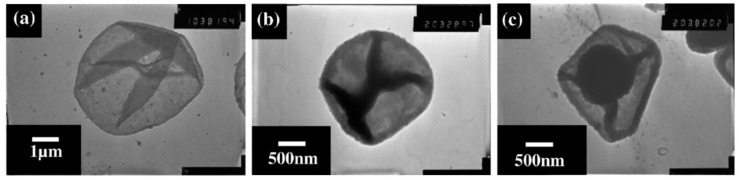
TEM images of (**a**) empty (PAH/PSS)_4_-PAH microcapsules, and those capsules loaded with (**b**) DNR and (**c**) DOX. Figure adapted with permission from Reference [93], copyright© 2006, Elsevier.

Moreover, the number of layers consisting the capsule shell can also affect the deposition of the drug within the capsules’ interior. Zhao et al. (2006) [93] reported a substantial decrease in the amount of DOX and DNR accumulating within poly(allylamine hydrochloride) (PAH)/PSS capsules consisting of 11 layers compared to that of 9 layers, as seen in Figure 4D. The addition of further polyelectrolyte layers onto the pre-formed microcapsules resulted in the loss of the pre-encapsulated PSS, via either loss during capsule washing stages or the charge neutralization with positively charged PAH. Consequently, this reduced extent of the drug loading within the capsules due to decreased drug affinity. Authors of this work demonstrate the accumulation of these drugs within the (PAH/PSS)_4_-PAH capsules through transmission electron microscopy (TEM) images, as illustrated in Figure 5. This is further evidenced by scanning force microscopy analysis, in which the average height of the capsule increases from ~16 nm (for empty capsules) to 310 and 740 nm for DNR- and DOX-loaded capsules, respectively.

## 3. The Release of LMW Drugs from PEMCs

### 3.1. Mechanism of Drug Release

The final step of successful drug delivery is the release of the drug to the diseased cell/tissue. The rate and extent of release of the loaded drug is crucial, and thus the release mechanisms of multiple vehicles have been studied in-depth within the literature. The release mechanism is again based upon the interactions between the polyelectrolyte that has been incorporated into the capsules, and the drug. In order for the liberation of the drug from the microcapsules to be achieved, the attractive interactions between the matrix material and the drug must be weakened. This may be achieved via changes in the local microenvironment of the drug-loaded system via introduction of external stimuli. Alterations in pH, ionic strength, and temperature can lead to liberation of the incorporated drug via changes in multilayer structure and stability. Moreover, depending upon the nature of the system, release and manipulation can be triggered via alternate means i.e., enzymatic degradation [18,94], light [83,95], ultrasound [96,97], or via magnetic field interactions (if magnetic nanoparticles are present [98,99,100]). Typically, a diffusion-limited release mechanism is observed for multilayer systems. For instance, the release of prednisolone from PAH/PSS-based capsules has been fitted to multiple release kinetic models with the first order and Higuchi models holding the finest fits. These fits suggest the mechanism of release is due to diffusion from the capsule core, or release through a porous matrix—hence the authors state diffusion of the drug is of crucial importance, as well as its dissolution [101].

**Figure 6:**
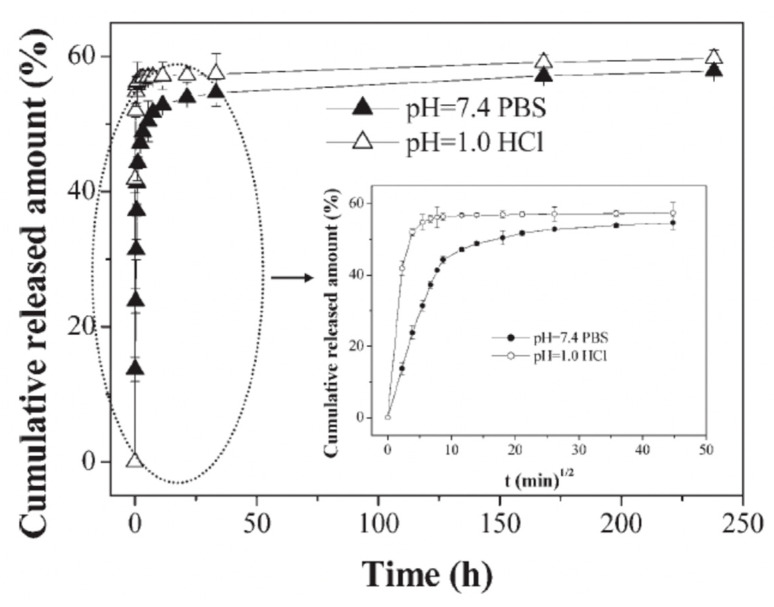
The effect of pH upon the cumulative release of DNR from ALG/CS-based microcapsules. The inset is of data collected during the first 40 h of incubation. The release was conducted in pH 7.4 PBS or 0.1 M HCl solution at 37 °C. Figure taken with permission from Reference [92], copyright© 2007, John Wiley and Sons.

In the case where the pre-incorporated matrix material is CMC, at lower pH values, the carboxylic ions of CMC become protonated and hence, become neutral. With consequence, this results in dramatically reduced drug affinity to the carboxylic groups of CMC (i.e., a weak CMC/drug complex) and eventually, the drug can ‘escape’ from the interior of the capsules via a diffusion mediated mechanism, this has been demonstrated using CS/ALG-based capsules. A quantitative illustration of the variation on pH values is shown in Figure 6. Initially, the drug is shown to be released at very fast rates in both cases (pH 7.4 and pH 1.0), this is the typical burst release mode. This is caused by the steep drug concentration gradient between the bulk and capsule interior. However, after some time, the release reaches a plateau [92].

**Figure 7:**
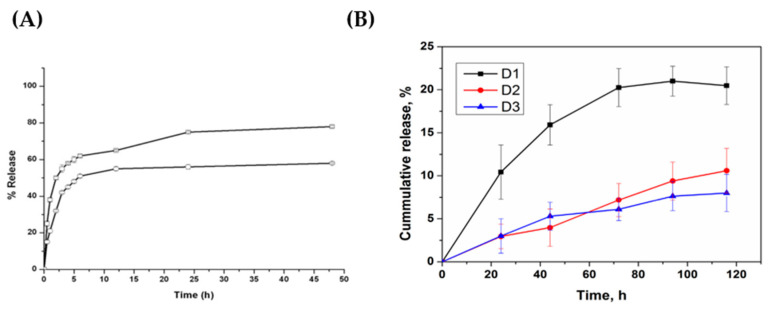
(**A**) The cumulative release of cisplatin from (ALG/PRO)_n_ capsules of 2 (open squares) and 3 (open circles) bilayers; (**B**) the cumulative release of DOX from DS/PARG-based capsules. D1: Initial and final capsule size of 550 nm, D2: Initial and final capsule size of 550 and 290 nm, respectively, and D3: Initial and final capsule size of 290 nm. All three sets of capsules shared an equal DOX feeding concentration, but were incubated for 1 h at either 25 °C or 90 °C for D1/D3, and D2, respectively. Figure A taken with permission from Reference [102], copyright© 2015, Elsevier. Figure B taken with permission from Reference [103], copyright© 2019, Elsevier.

Indeed, it can be assumed that an increased polymer density within the capsule matrix or shell will alter the release rate, also. It has been shown that the polymer density within a capsule system may be altered by increasing the polymer deposition time or via increasing the number of deposition steps. Vergaro et al. (2015) [102] demonstrated the effect of the number of polyelectrolyte layers forming the capsule upon the release rate of *cisplatin*. It was reported that the release rate was slowed when increasing the bilayer number from two to three within the (ALG/protamine sulfate (PRO))_n_ system, as illustrated by the release profiles illustrated in Figure 7A. This may be attributed to the thicker capsule shell, or increased polymer shell density, hindering the diffusion of *cisplatin* from the capsule interior. Furthermore, Trushina et al. (2019) [103] reported both the burst and sustained release modes of DOX from DS/PARG-based capsules. The sustained release was achieved via the heat-induced shrinkage of capsules prior to DOX release, resulting in compaction of the capsule and thus increasing the polymer density, slowing the DOX diffusion, these release profiles are also shown in Figure 7B.

**Figure 8:**
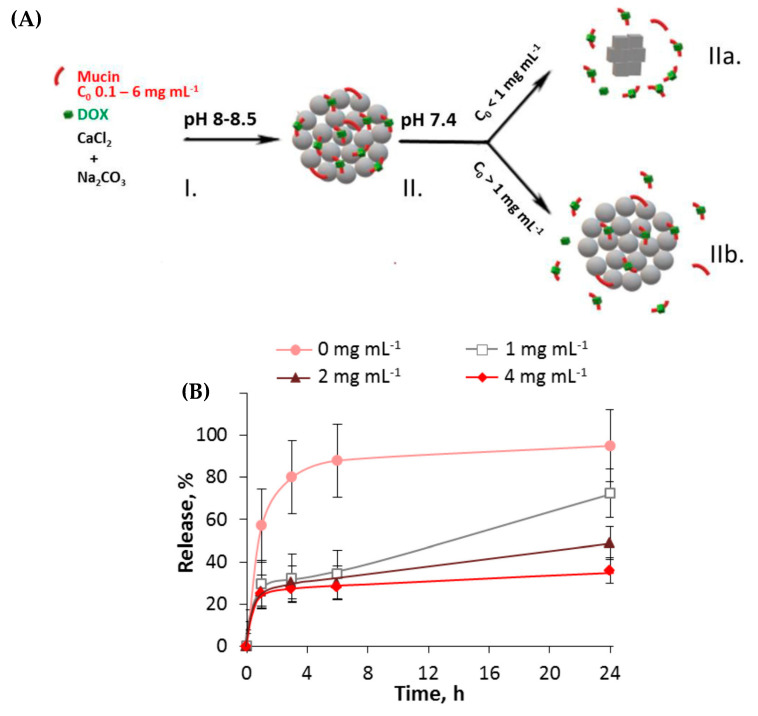
(**A**) Schematic illustration of (I) the co-synthesis of CaCO_3_-mucin hybrid crystals containing DOX and (II) the release of DOX at physiological conditions via diffusion of mucin-DOX complexes outwards due to re-crystallization of the CaCO_3_ crystals to calcite (IIa) and the complex diffusion out of the pores of the vaterite crystals (IIb). (**B**) The kinetics of DOX release from CaCO_3_-mucin-DOX hybrids at different mucin concentrations during co-synthesis. Figures taken with permission from Reference [80], copyright© 2019, Elsevier.

Balabushevich et al. (2019) [80,104] has recently developed hybrid CaCO_3_ crystals loaded with negatively charged mucin. Due to the matrix material of mucin, DOX can be easily incorporated into the vaterite crystals due to the strong electrostatic interactions present between the two molecules, as previously mentioned. Interestingly, the loading of mucin inside the vaterite crystals significantly alters the time taken for the vaterite crystals to re-crystallize to calcite (a thermodynamically stable, non-porous CaCO_3_ polymorph). At low concentrations of mucin present, a portion of the vaterite crystals re-crystallized to the stable calcite polymorph and DOX could be easily liberated, as represented in Figure 8(AIIa)–where DOX is released as a complex with mucin. However, at higher quantities of incorporated mucin, it was observed that the re-crystallization process of the vaterite crystals was inhibited (Figure 8(AIIb)). Hence, it is assumed that the drug release rate is controlled with respect of the mucin content in the hybrids, of which the release profiles demonstrate, also presented in Figure 8B. This study holds great implications for the controlled release of low molecular weight substances via simple tailoring of the matrix content within the CaCO_3_ crystals prior to coating, and perhaps may be incorporated into mucin-based multilayer capsules, as previously reported [105].

### 3.2. Effect of Multilayers upon the Kinetics of Drug Release

**Figure 9:**
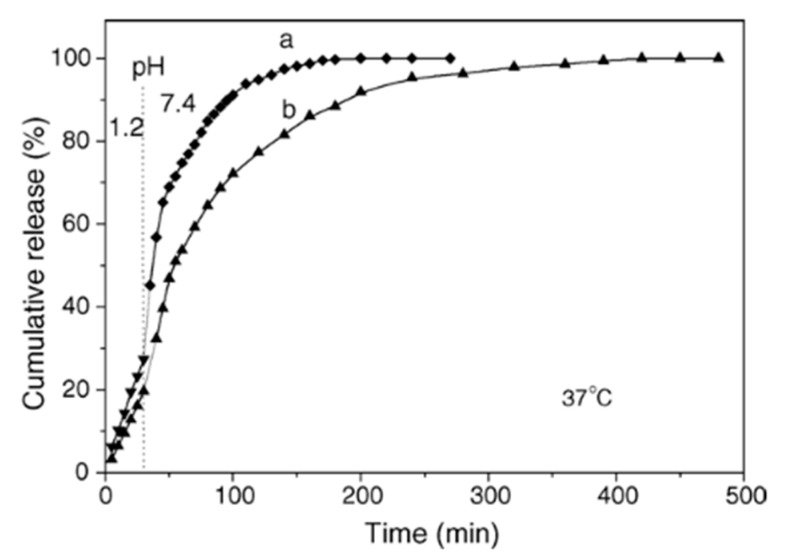
Release profiles of (**a**) IBU-loaded CaCO_3_ crystals and (**b**) IBU-loaded (PRO/PSS)_5_-coated CaCO_3_ crystals in simulated gastric fluid for the first 30 min, followed by simulated intestinal fluid for the remaining time, at 37 °C. Figure taken with permission from Reference [106], copyright© 2006, Elsevier.

Multilayers can have profound effects upon the release kinetics of vehicular systems. It has been established that, due to the presence of multilayers, the drug release rates may be hindered and hence, tuned. Wang et al. (2005) [106] compared the release dynamics of IBU-loaded bare-CaCO_3_ crystals and multilayer-coated CaCO_3_ crystals consisting of five PRO/PSS bilayers. This study was carried out in vitro via monitoring IBU release with UV-vis spectroscopy. The release mechanism was monitored for 30 min in a simulated gastric fluid (pH 1.2) environment, followed by simulated intestinal fluid (pH 7.4) at 37 °C. The resultant release profiles obtained from this study are illustrated in Figure 9. It is observed that the multilayer-coated crystals demonstrate a slower release rate (b) in comparison with the bare-CaCO_3_ microparticles (a), attributed to the hampered diffusion of the drug due to the additional multilayers.

**Figure 10:**
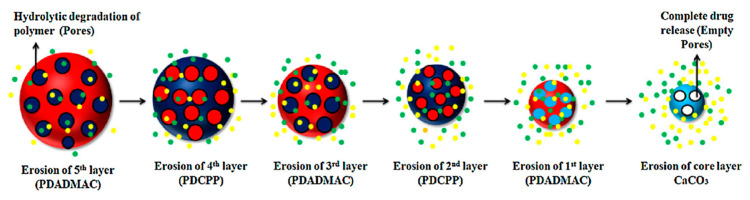
Schematic illustration of the hydrolytic degradation of PDADMAC)/PDCPP) multilayers, leading to the release of both cisplatin and chrysin. Schematic taken with permission from Reference [107], copyright© 2018, Elsevier.

Moreover, a diffusion-mediated mechanism of release for PDADMAC/poly [di(sodium carboxyphenoxy)phosphazene] (PDCPP) coated CaCO_3_ nanoparticles was confirmed by Mehnath et al. (2018) [107]. The coating possesses hydrophilic properties and undergoes hydrolytic degradation (Figure 10); hence, pores form within the shell form and the matrix swells, leading to enhanced chrysin and *cisplatin* diffusion outwards. This mechanism of release results in a burst-release mode, followed by a sustained release of both drugs, with the stability of the polymer complex playing a crucial role in the control of the release rate. This suggests the release rate of this system may be tailored through minor changes in the local microenvironment (i.e., pH or ionic strength) to alter the initial strength of the polymer complex. Moreover, multilayers deposited via the LbL approach hold the ability to slow the rate of drug release and provide a more sustained release-like profile in comparison with the absence of multilayers, where rapid release is typically observed. Strong binding of small and large charged molecules to free (uncompensated by polyelectrolytes) changes can be evaluated by mobility of the laden molecules and the binding strongly affects the release kinetics [12,108,109].

### 3.3. Biological Applications of LMW Drug Loaded PEMCs

**Figure 11:**
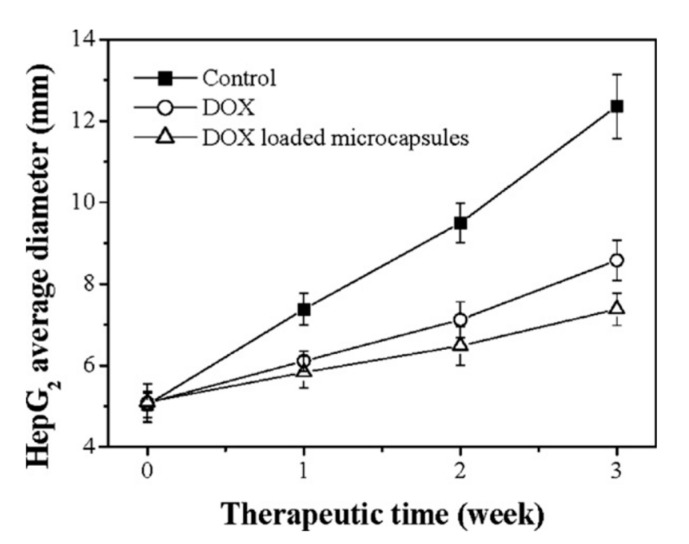
The diameter of HepG2 BALB/c/nu tumors measured over the three-week period via use of encapsulated and free DOX treatments. The capsules utilized are CS/ALG-based, templated from CMC-doped CaCO_3_ crystals. Figure taken with permission from Reference [82], copyright© 2007, Elsevier.

In vivo experiments have been carried out to monitor the growth of tumor cells in the presence of anticancer DOX-loaded multilayer capsules over time. Zhao et al. (2007) [82] reported the effect of such capsules upon HepG2 BALB/c/nu tumors in mice. Eighteen mice of similar weight were used for this experiment. A control was established, with two other conditions: mice injected with free DOX, and those injected with encapsulated DOX (CS/ALG-based capsules templated on CMC-doped CaCO_3_ crystals). Both free and encapsulated DOX drug were injected into the tumor once a week for three weeks. The diameters of HepG2 tumors for the three conditions are summarized in Figure 11. It can be clearly observed that DOX loaded microcapsules inhibit the growth of tumor more effectively than free DOX. Thus, it can be suggested that loaded PEMCs hold better efficacy as an antitumor treatment in comparison with the free DOX, even when not all of the drug is time-released from the microcapsules. Moreover, Han et al. (2008) [92] reported a similar study but utilized the anticancer DNR drug on the BEL-7402 tumor cell line. Again, while using tumors without any treatment as the control, they simultaneously tested tumors that were injected with free DNR and DNR-loaded microcapsules once a week for three weeks. Again, the significant reduction of the diameter of tumor cells in the third week is prevalent, especially those tumors treated with DNR-loaded microcapsules.

A great reduction in tumor volume was also reported by Mehnath et al. (2018) [107]. Tumor-induced hamsters were orally given CaCO_3_ crystals coated with PDADMAC/PDCPP layers dual-loaded with chrysin and *cisplatin.* Those tumors treated with dual-loaded crystals exhibited volume regressions of 92% as opposed to those treated with *cisplatin* alone. Similarly, 290 nm shrunken DOX-loaded (DS/PARG)_3_ capsules have shown to accumulate in human breast adenocarcinoma cells in vitro, including DOX resistant MCF-7/ADR cells, overcoming the drug resistance and reducing cell viability [103]. Moreover, identically sized capsules consisting of the same polymers were loaded with the low molecular weight chemotherapeutic drugs; gemcitabine and chlodronate. Following intravenous administration, it was reported the capsules favor uptake into the liver and lungs, showing uptake into macrophages and epithelial cells. Furthermore, when compared to healthy lungs, the capsules had higher accumulation rates into tumor lungs. The efficacy of this lung cancer treatment was confirmed via the reduced viability of lung cancer cells and the inhibition of the tumor-promoting function of bone-marrow derived macrophages by gemcitabine and chlodronate, respectively [110]. Overall, (DS/PARG)_n_ capsules show great potential for the passive treatment of the cancer of the lung, as well as the prospective treatment of many cancers due to their ability to overcome drug resistance in their shrunken state.

## 4. Summary

The successful loading of LMW drugs is demonstrated in LbL multilayer-coated CaCO_3_ crystals, as well as micro- and nanosized PEMCs templated onto the crystals. The two most prevalent methods of loading were discussed; namely the spontaneous and charge-controlled attraction and repulsion processes. The spontaneous deposition process is a powerful and effective tool for the encapsulation of such LMW drugs, even at lower feeding concentrations when the loading is not thermodynamically saturated. Mesoporous CaCO_3_, being able to host a wide variety of molecules, can host a plethora of matrix-materials and so may have large scope to host a large variety of small drugs effectively. These systems have shown to be widely tailorable; such that factors including the drug-particle incubation temperature, ionic strength, the number of polyelectrolyte layers, and drug feeding concentration have shown to have a strong influence on the uptake of drugs into the respective capsule systems. Moreover, the release mechanisms of such small drugs have also been discussed, namely diffusion-mediated modes of release. It was found that varying several environmental properties can profoundly affect the release rate of these small drugs. The change of the pH value can tailor the release, especially in these polymer-based systems. Pre-incorporated polyelectrolyte ionized under certain pH conditions can result in the weakening or the destruction of the polyelectrolyte-drug complex and initiate a diffusive release. The effect of multilayers themselves have shown to be robust methods of slowing the release rates of multiple drugs, typically hampering their diffusion. This suggests that PEMCs are excellent candidates for the sustained release of LMW drugs. Finally, both in vitro and in vivo studies have established the efficacy of anticancer drug-loaded CaCO_3_ vaterite crystals and multilayer templated capsules on the treatment of tumors cells, in contrast with the free drug itself. Overall, such PEMCs have shown great promise in the hosting and the controlled and targeted release of LMW drugs; easily tuned through choice of the polyelectrolytes themselves, the matrix material and local microenvironmental changes. Nevertheless, studies are continuously being carried out in search for the optimization of such popular PEMC systems, through the control of multilayer properties in order to achieve sustained release in accordance with the drug dosage necessary.
